# Loss-of-Function Piezo1 Mutations Display Altered Stability Driven by Ubiquitination and Proteasomal Degradation

**DOI:** 10.3389/fphar.2021.766416

**Published:** 2021-11-19

**Authors:** Zijing Zhou, Jinyuan Vero Li, Boris Martinac, Charles D. Cox

**Affiliations:** ^1^ Molecular Cardiology and Biophysics Division, Victor Chang Cardiac Research Institute, Sydney, NSW, Australia; ^2^ St. Vincent’s Clinical School, Faculty of Medicine, University of New South Wales, Sydney, NSW, Australia

**Keywords:** post-translational modification (PMT), protein biosynthesis, mechanosensation, ubiquitiantion, proteasomal degradation

## Abstract

Missense mutations in the gene that encodes for the mechanically-gated ion channel Piezo1 have been linked to a number of diseases. Gain-of-function variants are linked to a hereditary anaemia and loss-of-function variants have been linked to generalized lymphatic dysplasia and bicuspid aortic valve. Two previously characterized mutations, S217L and G2029R, both exhibit reduced plasma membrane trafficking. Here we show that both mutations also display reduced stability and higher turnover rates than wild-type Piezo1 channels. This occurs through increased ubiquitination and subsequent proteasomal degradation. Congruent with this, proteasome inhibition using *N*-acetyl-l-leucyl-l-leucyl-l-norleucinal (ALLN) reduced the degradation of both mutant proteins. While ALLN treatment could not rescue the function of S217L we show via multiple complementary methodologies that proteasome inhibition via ALLN treatment can not only prevent G2029R turnover but increase the membrane localized pool of this variant and the functional Piezo1 mechanosensitive currents. This data in combination with a precision medicine approach provides a new potential therapeutic avenue for the treatment of Piezo1 mediated channelopathies.

## Introduction

Mechanosensitive (MS) ion channels are a structurally diverse class of cellular sensors that decode mechanical cues (Martinac and Cox, 2017; Cox et al., 2019; Poole, 2021; Syeda, 2021). The mechanosensitive channel Piezo1 has an emerging role in cardiovascular biology (Li et al., 2014; Rode et al., 2017; Douguet et al., 2019; Jiang et al., 2021; Yu et al., 2021) and is central in force sensing by vascular endothelial cells (Li et al., 2014; Ranade et al., 2014; Rode et al., 2017; Albarrán-Juárez et al., 2018; Nonomura et al., 2018). This channel responds to both membrane tension (Lewis and Grandl, 2015; Cox et al., 2016; Syeda et al., 2016) and shear stress (Ranade et al., 2014; Albarrán-Juárez et al., 2018; Maneshi et al., 2018; Lai et al., 2021) (whether these are separate molecular mechanisms remains to be determined) and is important in the development of valves in different tissues including the lymphatic system (Nonomura et al., 2018) and heart (Duchemin et al., 2019; Faucherre et al., 2020). Since its discovery and cloning (Coste et al., 2010) missense variants in the *PIEZO1* gene have been linked to several human pathologies (Zarychanski et al., 2012; Albuisson et al., 2013; Bae et al., 2013; Lukacs et al., 2015; Glogowska et al., 2017; Murthy et al., 2017; Faucherre et al., 2020). In particular, loss-of-function missense variants in *PIEZO1* have been linked to generalized lymphatic dysplasia (Fotiou et al., 2015; Lukacs et al., 2015) and bicuspid aortic valve (Faucherre et al., 2020).

Like many other integral membrane proteins, Piezo1 channels undergo biosynthetic quality control in the endoplasmic reticulum (ER) (Tannous et al., 2015). During this process Piezo1 undergoes significant N-linked glycosylation, the process by which oligosaccharides are covalently attached to asparagine residues, which fulfils a critical role in its biosynthetic quality control (Li et al., 2020). These N-glycans may act as molecular tethers and participate in force sensing (particularly shear stress sensing) as shown for the epithelial sodium channel ENaC (Knoepp et al., 2020). Previously, we showed that trafficking defective Piezo1 mutations (disease linked or from structure function studies (Vero Li et al., 2021)) display modified N-linked glycosylation (Li et al., 2021). Specifically, we noted that loss-of-function variants of Piezo1 such as S217L and G2029R show reduced N-linked glycosylation. Using the extensive published information from the K_v11.1_ channel literature we attempted through low temperature and pharmacological means (Robertson and January 2006; Smith et al., 2013; Anderson et al., 2014) to rescue surface expression and hence function of Piezo1 mutants but neither approach was successful.

As the first step of post-translational quality control, mis-folded proteins are recognised and sent to the proteasome for degradation by a process termed ER-associated degradation or ERAD. ERAD is mostly, if not fully, dependent on the ubiquitin-proteasome system (UPS) (Ron and Walter, 2007; Christianson and Ye, 2014). The UPS represents the major quality-control machinery for guaranteeing the normal function of many proteins via eliminating misfolded protein products. Furthermore, shutting down the UPS using proteasome inhibitors can help trafficking-deficient mutants to escape ERAD and rescue the function of proteins such as; Msh2 (Arlow et al., 2013), RESA1 (Mohanraj et al., 2019) or Na_v_1.1 (Rusconi et al., 2007), both *in vitro* and *in vivo*.

In the current study, we confirmed that the disease-causing mutants S217L and G2029R of *PIEZO1* are both trafficking-deficient. Moreover, we showed that both mutants exhibited ER retention, reduced protein stability and higher levels of ubiquitination, all of which are the hallmarks of ERAD. Inhibiting proteasomal degradation using ALLN reduced turnover of the mutant proteins. Importantly, the protein expression of G2029R was also stabilized using the clinically approved proteasome inhibitor Bortezomib (Chen et al., 2011). Finally, we showed that proteasome inhibition rescued the membrane targeting and increased stretch-activated currents in cells expressing G2029R. This raises the possibility that clinically available proteasome inhibitors could be used in conjunction with a precision medicine approach to treat a subset of loss-of-function *PIEZO1* mutations.

## Materials and Methods

### Antibodies

The following commercially available antibodies were used: anti-GFP antibody (Santa Cruz Biotechnology, Dallas, TX, United States; 1:5,000 dilution); mouse monoclonal anti-Piezo1 antibody (Cat# NBP2-88938, Novus Biologicals, Centennial, CO, United States; 1:1,000 dilution); mouse anti-α-actinin antibody (Santa Cruz Biotechnology; 1:5,000 dilution); mouse anti-Na^+^/K^+^ ATPase antibody (a6F, DSHB; 1:1,000 dilution); mouse anti-ubiquitin antibody (clone FK2, ENZO, 1:1,000); rabbit anti-GAPDH antibody (CellSignaling, 1:1,000); anti-rabbit IRDye680 (Li-Cor, 1:20,000); anti-mouse IRDye800 (Li-Cor, 1:20,000); mouse anti-HA antibody (Sigma, 1: 200 for IF); Alexa Fluor 555 anti-mouse (ThermoFisher, 1:200 for IF). Unless specified, all the antibody dilutions refer to western blotting.

### Cell Culture and Transfection

Piezo1^−/−^ HEK293T cells (Lukacs et al., 2015) were a gift from Dr. Ardem Patapoutian (The Scripps Research Institute, La Jolla, CA, United States); HeLa cells were purchased from ATCC (Cell lines were not authenticated and were not listed in the database of commonly misidentified cell lines maintained by ICLAC (http://iclac.org) and NCBI Biosample (http://www.ncbi.nlm.nih.gov/biosample). All cell lines were confirmed to be mycoplasma free. HEK cells were transfected with polyethylenimine (PEI); HeLa cells were transfected with Lipofectamine 3,000 transfection reagent (ThermoFisher Scientific).

### Ubiquitination Assay

Ubiquitination assay protocol is modified from previous work (Zhang et al., 2017; Cao et al., 2021). WT or mutant Piezo1 transfected cells were lysed in the co-IP buffer (1% w/v CHAPS, 0.6% w/v soy PC, 140 mM NaCl, 1 mM EDTA, 25 mM NaPIPES) supplemented with 2 mM 1,4-Dithiothreitol (Sigma), 1× protease-inhibitor cocktail (Roche) and 20 mM N-ethylmaleimide (E3876; Sigma), and the lysates were centrifuged at 16,000 ×g for 10 min at 4°C; the expression of each protein of interest was confirmed by immunoblotting 5% of the collected supernatants, and the remaining supernatants were incubated with 0.6 μg of anti-GFP antibody and 10 μL of Protein G Dynabeads (ThermoFisher) at 4°C overnight. The recovered beads were washed three times with the co-IP wash buffer (25 mM NaPIPES, 140 mM NaCl, 0.6% w/v CHAPS, 0.14% PC) supplemented with 2 mM 1,4-Dithiothreitol (Sigma), 1× protease-inhibitor cocktail (Roche) and 20 mM N-ethylmaleimide (E3876; Sigma), and heated in 2x sodium dodecyl sulphate (SDS)-PAGE loading buffer containing 1 M urea and 10 mM tris(2-carboxyethyl)phosphine (TCEP) at 62°C for 5 min, and the eluted proteins were immunoblotted with anti-GFP or anti-ubiquitin antibodies.

### Live Cell Labelling

For live cell membrane protein labelling, Piezo1^−/−^ HEK293T cells were plated on 96 well clear bottom plate (ThermoFisher) coated with 0.1 mg/ml of Poly-L-Lysine (Sigma). Cells were transfected with pIRES-GFP constructs carrying WT or mutant Piezo1 (125 ng cDNA per well) with PEI. Sixty to 72 h after transfection, live labelling was performed by incubating the cells with anti-HA (Sigma, 1:100) antibody for 20 min at 37°C. The cells were then washed with DMEM six times before being incubated with Alexa Fluor 555 anti-mouse secondary antibody (1:200) for 15 min at room temperature (22°C). Cells were washed again 5 times with DMEM and twice with phosphate buffered saline (PBS), then fixed with 4% paraformaldehyde (PFA) for 20 min at room temperature. PFA was washed off and replaced with PBS before confocal analysis.

For co-labelling of Piezo1 and the endoplasmic reticulum (ER), HeLa cells were transfected with WT or mutant Piezo1 fused with GFP. After transfection for 60–72 h, cells were washed with PBS twice then incubated with 1 μM of ER-Tracker Red dye (Invitrogen) for 20 min at 37°C. The cells were washed again with PBS three times and fixed with 4% PFA for 20 min at room temperature. PFA was then replaced with PBS, and the ER or Piezo1-GFP signals were visualized using confocal microscopy (Zeiss LSM 700 inverted).

### Proteasomal and Lysosomal Inhibitor Treatment

Piezo1^−/−^ HEK293T cells were transfected with GFP fused WT or mutant Piezo1 before treatment. The transfected cells were treated with the following conditions: 10 μg/ml of CHX (Sigma) for 4 or 8 h; 10 mM of NH_4_Cl (Sigma) or 10 μM of ALLN (Sigma) for 9 or 24 h; 1 or 10 nM of Bortezomib (BTZ) for 24 h. The cells were then lysed in radioimmunoprecipitation assay (RIPA) buffer (150 mM NaCl, 1.0% w/v NP40, 0.5% w/v sodium deoxycholate, 0.1% w/v SDS, 50 mM Tris, pH 7.4) supplemented with 1× protease-inhibitor cocktail and 10 mM tris(2-carboxyethyl)phosphine (TCEP). Cell lysates were subjected to western blotting.

### Electrophysiology

Transiently transfected Piezo1^−/−^ HEK293T cells were plated on 35 mm dishes for patch clamp analysis. The extracellular solution for cell-attached patches contained high K^+^ to zero the membrane potential; it consisted of 90 mM potassium aspartate, 50 mM KCl, 1 mM MgCl_2_ and 10 mM HEPES (pH 7.2) adjusted with 5 M KOH. The pipette solution contained 140 mM CsCl with 10 mM HEPES (pH 7.2) adjusted with the respective hydroxide. Ethylene glycol-bis(β-aminoethyl etlier)-N,N,N′,N′-tetraacetic acid (EGTA) was added to control levels of free pipette (extracellular) Ca^2+^ using the online EGTA calculator—Ca-EGTA Calculator TS v1.3—Maxchelator. Negative pressure was applied to patch pipettes using a High-Speed Pressure Clamp-1 (ALA Scientific Instruments, Farmingdale, NY, United States) and recorded in millimetres of mercury (mmHg) using a piezoelectric pressure transducer (WPI, Sarasota, FL, United States). Borosilicate glass pipettes (Sigma-Aldrich) were pulled with a vertical pipette puller (PP-83, Narashige, Tokyo, Japan) to produce electrodes with a resistance of 1.8–2.2 MΩ. The Piezo1 currents were amplified using an AxoPatch 200B amplifier (Axon Instruments, Union City, CA, United States), and data were sampled at a rate of 10 kHz with 1 kHz filtration and analysed using pCLAMP10 software (Axon Instruments).

### Western Blotting

Transiently transfected cells were solubilized in a modified RIPA buffer [Tris buffer 10 mM, ethylenediaminetetraacetic acid (EDTA) 1 mM, NaCl 140 mM, in (% w/v): Sodium deoxycholate 0.1, SDS 0.1, Triton X-100 1.0, pH 7.2] supplemented with 1× EDTA-free protease inhibitor cocktail tablets (Sigma-Aldrich), 1 mM (phenylmethylsulfonyl fluoride) PMSF, 10 mM TCEP, and 1 mM N-ethylmaleimide (NEM) for 10 min on a rotating wheel at 4°C. Cell lysates were cleared by centrifugation at 13,000 ×g at 4°C for 10 min. For quantitative western blot analysis, GFP-fused Piezo1 was probed with a rabbit monoclonal anti-GFP antibody (Santa Cruz Biotechnology, Dallas, TX, United States); or a mouse monoclonal anti-Piezo1 antibody (Cat# NBP2-88938, Novus Biologicals, Centennial, CO, United States); mouse anti-α-actinin antibody (Santa Cruz Biotechnology) was added simultaneously for a loading comparison followed by anti-rabbit IRDye680 and anti-mouse IRDye800 (Li-Cor) to enable quantification with the LI-COR Odyssey system (LI-COR Biotechnology, Lincoln, NE, United States).

### Biotinylation

For each well in a 6-well plate, transiently transfected cells were washed three times using ice cold PBS and then incubated with biotin buffer (154 mM NaCl, 10 mM HEPES, 3 mM KCL, 1 mM MgCl_2_, 0.1 mM CaCl_2_, 10 mM glucose, pH 7.6) containing 1 mg/ml Sulfo-NHS-Biotin (ThermoFisher Scientific, EZ-Link™, Lot. No. TI266926) for 1 h on ice. The biotin buffer was then washed off and quenched using 50 mM of Glycine in PBS for 10 min on ice. Cells were then washed three times using ice cold PBS and solubilized using 250 μL RIPA buffer. Cell lysates were cleared by centrifugation at 13,000 ×g at 4°C for 10 min 20 μL supernatant was taken and supplemented with 2% w/v SDS and 0.8 M urea and designated as the “input” sample. The remaining supernatant was incubated overnight at 4°C with 20 μL Streptavidin-Agarose beads (Sigma-Aldrich, Lot#: SLBR5741V) blocked using 0.5% w/v bovine serum albumin for 1 h. Beads were collected and washed three times using RIPA buffer. For protein elution, beads were loaded with 20 μL 0.9% w/v NaCl solution supplemented with 2% SDS and 0.8 M urea, and heated at 62°C for 5 min. Then they were centrifuged at 500 ×g at 4°C for 1 min in the column, and the biotinylated eluate was collected. Identical protein amounts from input and biotinylated samples were loaded for western blotting then probed with; mouse monoclonal anti-Piezo1 antibody (Cat. No. NBP2-75617, Novus Biologicals), rabbit anti-GAPDH (CellSignaling) and mouse anti-Na^+^/K^+^ ATPase antibody (a6F, DSHB).

### Statistics

Protein bands in western blots were quantified using ImageJ software, and peak currents were measured using Clampfit software. All data are expressed as means ± SEM; n represents the number of independent biological replicates. *p* < 0.05 was considered statistically significant.

## Results

### S217L and G2029R Piezo1 Are Less Stable Compared to Wild-type Piezo1

**FIGURE 1:**
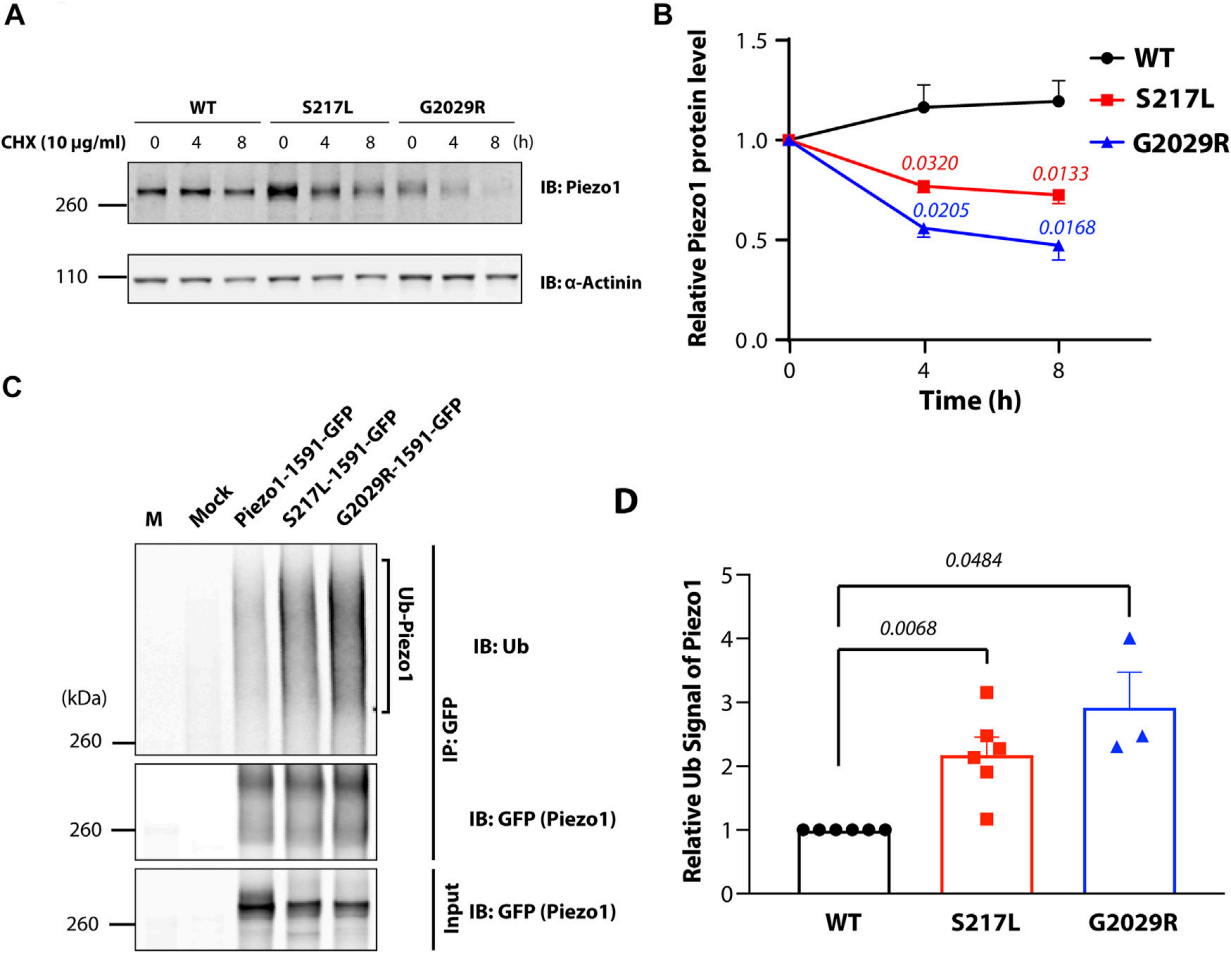
Instability of Piezo1 loss-of-function mutants correlates with increased ubiquitination. **(A)** Piezo1^−/−^ HEK293T cells overexpressing wild-type (WT) or mutant Piezo1-GFP were treated with 10 μg/ml cycloheximide (CHX) for 0, 4 or 8 h before being collected for western blotting. **(B)** Summary data of panel **(A)** showing Piezo1 protein levels normalized to α-Actinin. (*p = 0.0320 and *p = 0.0133 for S217L at 4 and 8 h; *p = 0.0180 and *p = 0.0168 for G2029R at 4 and 8 h) **(C)** WT or mutant Piezo1-GFP expressed in Piezo1^−/−^ HEK293T cells were immunoprecipitated using an anti-GFP antibody and the eluted fraction and input cell lysate were probed for Piezo1 levels. An immunoblot of the immunoprecipitated protein using a mono- and poly-ubiquitin (Ub) antibody is also shown (M = marker). **(D)** Summary data of panel C showing the level of Piezo1 ubiquitination normalized to the level of immunoprecipitated Piezo1 protein (*n* = 4 for all experiments). One way ANOVA with Dunnett’s multiple comparison test was used for statistical analysis.

S217L and G2029R Piezo1 are both loss-of-function mutants (Faucherre et al., 2020; Lukacs et al., 2015; Li et al., 2020). The exact mechanisms that underlie this loss-of-function have not been fully explored. The cycloheximide chase procedure has permitted visualization of the degradation kinetics of the steady state population of a variety of cellular proteins (Kao et al., 2015). To understand the turnover rate of Piezo1 channels, we applied cycloheximide (CHX) to the culture media of Piezo1^−/−^ HEK293T cells expressing wild-type (WT) or mutant Piezo1 (S217L and G2029R) fused to GFP (Cox et al., 2016; Li et al., 2020; Vero Li et al., 2021; Ridone et al., 2020; Buyan et al., 2020). The protein level of WT Piezo1 was stable and did not decrease even after 8 h treatment with 10 μg/ml of CHX. In comparison, both S217L and G2029R showed significantly reduced protein amounts after 4- or 8-h treatment compared to untreated controls. Specifically, we observed a 27 or 53% reduction in protein level for S217L and G2029R, respectively, after 8-h treatment, implying a faster turnover rate of the mutant proteins (Figures 1A,B). One potential mechanism that could drive faster protein turnover is ubiquitination. Protein ubiquitination is one of the key processes in endoplasmic reticulum associated degradation (ERAD) enabling the elimination of mis-folded proteins (Ron and Walter, 2007; Christianson and Ye, 2014). As a result, we examined the ubiquitination levels of Piezo1 and mutant Piezo1 proteins expressed in Piezo1^−/−^ HEK293T cells. To do this, we immunoprecipitated overexpressed Piezo1 and analysed the ubiquitination signal specific to Piezo1 in the elution (Figure 1C). The normalized ubiquitination levels of both S217L and G2029R were 2.2 and 2.8-fold higher respectively than WT. Interestingly, the GLD linked mutant protein G2029R seemed to have a higher ubiquitination level than that of S217L (Figures 1C,D).

### Degradation of S217L and G2029R Piezo1 Is Proteasome Dependent

**FIGURE 2:**
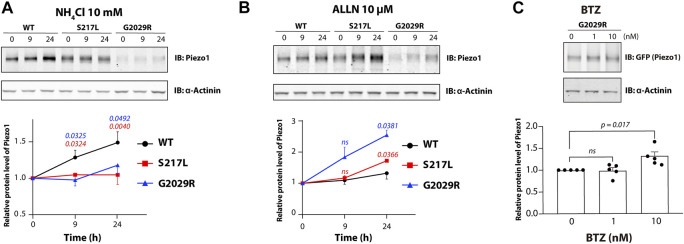
S217L and G2029R Piezo1 mutants go through proteasomal degradation. Piezo1^−/−^HEK293T cells overexpressing WT or mutant Piezo1 were treated with **(A)** a lysosomal degradation inhibitor NH_4_Cl or **(B)** a proteasomal degradation inhibitor ALLN for 0, 9 or 24 h. Representative results are shown in the upper panel. Lower panel, summary data of the corresponding experiments with comparison between mutant and WT Piezo1 shown in blue for G2029R and in red for S217L. **(C)** Piezo1^−/−^ HEK293T cells overexpressing G2029R Piezo1 were treated with 0, 1 or 10 nM of Bortezomib for 24 h before being collected for western blot. Upper panel, representative western blot. Lower panel, summary data of the western blot experiments. One way ANOVA with Dunnett’s multiple comparison test was used for statistical analysis (ns = not significant).

Proteasome dependent degradation, in addition to ubiquitination, is another hallmark of ERAD. To investigate the degradation pathway for WT and mutant Piezo1, we utilized NH_4_Cl a generic inhibitor of lysosomal degradation and a non-specific inhibitor of proteasomal degradation acetyl-l-leucyl-l-leucyl-l-norleucinal (ALLN). Treatment with 10 mM of NH_4_Cl caused an increase in the levels of WT Piezo1 protein. Relative WT Piezo1 protein levels were increased by 49% after treatment for 24 h. This is consistent with WT Piezo1 being degraded by a lysosomal pathway. In contrast NH_4_Cl had little effect on the protein levels of S217L and G2029R (Figure 2A). In contrast to that, both S217L and G2029R showed significantly increased protein expression (72 and 155% increase in protein level respectively for 24-h treatment) in response to ALLN treatment (Figure 2B). Importantly, the level of G2029R protein expression increased more than that of S217L, which was consistent with the higher level of G2029R ubiquitination and faster turnover rate determined using CHX treatment (Figures 1B,D). We further asked whether the ability of ALLN to reduce G2029R protein turnover could be recapitulated using a specific proteasome inhibitor. To test this, we used the clinically available anti-cancer agent Bortezomib (BTZ) and treated Piezo1^−/−^ HEK293T which overexpressed the G2029R mutant with concentrations that ranged from 0.1–10 nM. The protein level of the G2029R mutant was significantly increased after 24 h treatment with BTZ at 10 nM when compared to untreated controls (Figure 2C). Collectively our data indicates that both S217L and G2029R Piezo1 go through ER associated degradation when overexpressed in HEK293T cells.

### Aberrant Membrane Targeting of S217L and G2029R Piezo1

**FIGURE 3:**
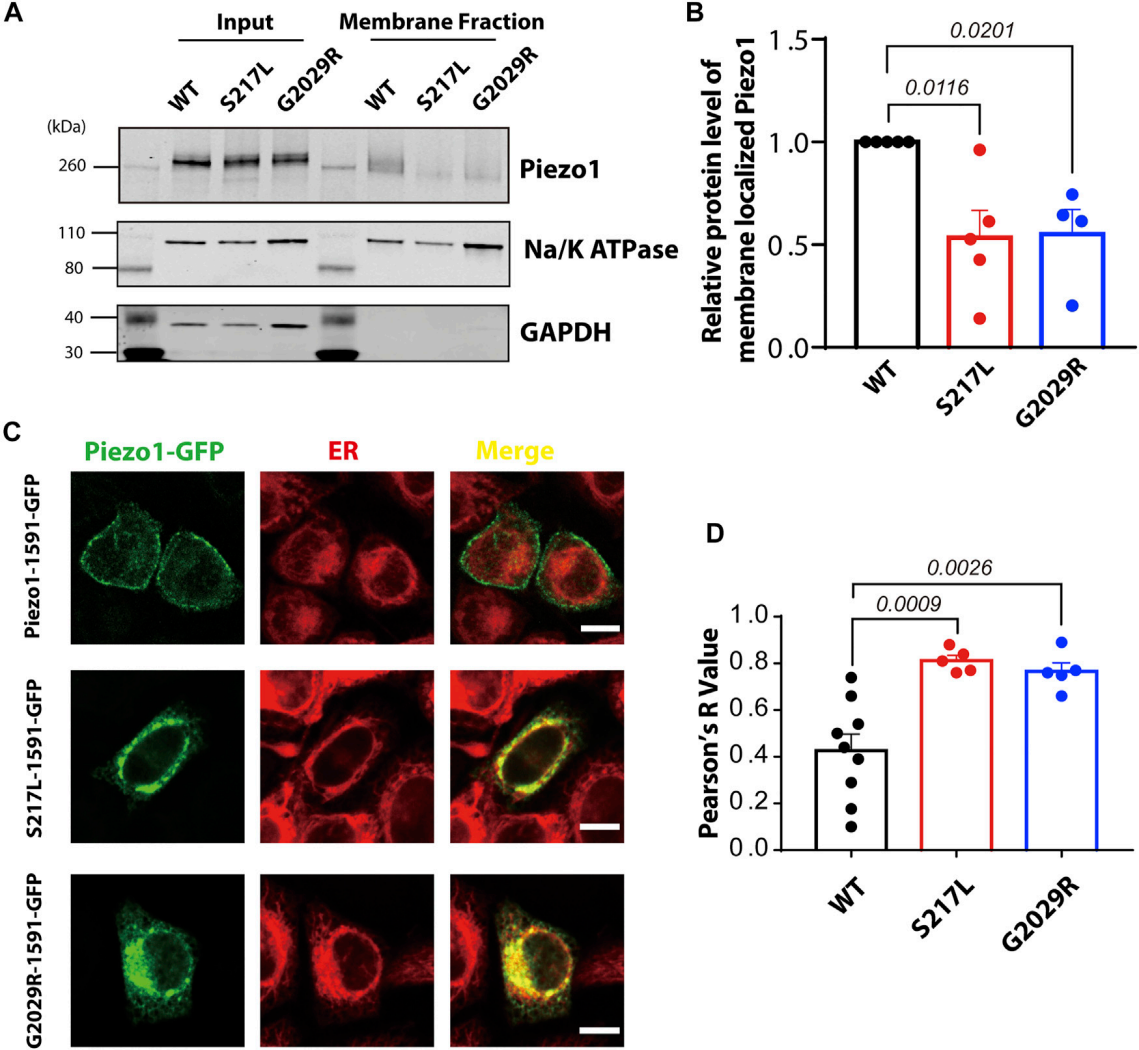
S217L and G2029R are trafficking-deficient mutants. **(A)** Western blot showing the membrane localized fraction of WT and mutant Piezo1 proteins Piezo1 from Piezo1^−/−^ HEK293T cells using a standard biotinylation assay. The membrane fraction of the eluted lysate is visualised using the ubiquitous membrane protein Na^+^/K^+^ ATPase and the cytosolic protein GAPDH is shown as a comparison for whole protein lysate (Input) and bead elution (Membrane Fraction). **(B)** Summary data of panel **(A)**. One way ANOVA with Dunnett’s multiple comparison test was used for statistical analysis. **(C)** HeLa cells overexpressing WT or mutant Piezo1 with GFP tag were stained with ER tracker and analysed using confocal microscopy. Notice that the WT Piezo1 is localized to the plasma membrane while the mutant Piezo1 co-localizes almost exclusively with the ER tracker. Scale bar, 10 µm. **(D)** Summary data showing the overlap of GFP (Piezo1) and the ER signal indicated by Pearson’s R value. One way ANOVA with Dunnett’s multiple comparison test was used for statistical analysis.

Mis-localization of G2029R has been previously reported (Lukacs et al., 2015) and replicated (Li et al., 2020). However, conflicting reports exist regarding the sub-cellular localization of S217L (Faucherre et al., 2020; Li et al., 2020). To investigate the membrane targeting efficiency of these two mutants, we performed membrane biotinylation by biotinylating the membrane proteins and pulling down those proteins with streptavidin conjugated agarose beads. The Piezo1 signal in the membrane fraction for the two mutants, normalized to Na^+^/K^+^-ATPase and input Piezo1, was significantly reduced compared to WT Piezo1 by >50% (Figures 3A,B), suggesting aberrant trafficking and inefficient membrane targeting of both S217L and G2029R. To further corroborate these observations, we studied the localization of WT and mutant Piezo1 by overexpressing GFP fused Piezo1 in HeLa cells, and then imaged live cells stained with a marker for the ER using confocal microscopy. Unlike the WT Piezo1, which was largely localized to the plasma membrane, S217L and G2029R Piezo1 were both largely confined to the ER indicated by the high level of co-localization with the signal from the ER tracker (Figure 3C). We quantified the degree of co-localization between the Piezo1-GFP fusion proteins and the ER tracker and observed that the co-localization co-efficient for WT Piezo1 with the ER tracker was >1.8 fold less than that for the mutant proteins (Figure 3D).

### ALLN Is Able to Rescue Membrane Localization of G2029R Piezo1

**FIGURE 4:**
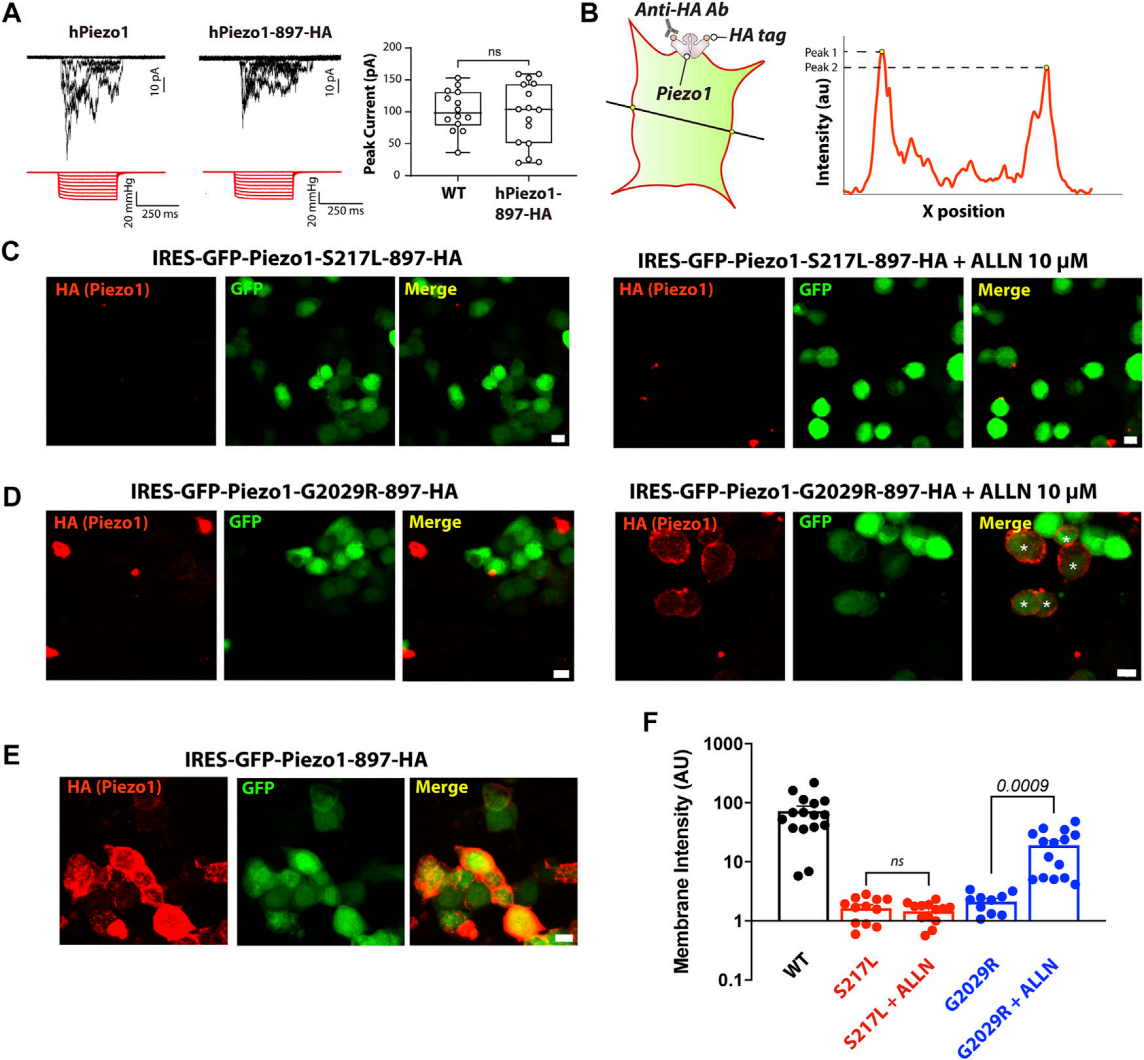
ALLN partially rescues the membrane-localization of G2029R. **(A)** Piezo1^−/−^ HEK293T cells overexpressing WT or Piezo1-897HA were subjected to cell-attached patch clamping. Representative traces in response to square wave negative pressure pulses are shown. The right panel shows the summary data of peak currents elicited from cell-attached patches as a box and whiskers plot illustrating the minimum and maximum. **(B)** Schematic showing how the membrane intensity is calculated. A straight line was drawn across the GFP positive cells with only the GFP channel visible to preclude bias. The peak value of the HA signal within the membrane region, determined by GFP channel and bright field, was determined using *Image J*’s *Plot Profile* tool. Peak values on each side of the cell were averaged as one data point. **(C–E)** Piezo1^−/−^ HEK293T cells overexpressing WT or mutant Piezo1 with an extracellular 897-HA tag were stained with anti-HA antibody and Alexa-555 goat anti mouse secondary antibody before PFA fixation. All figures were taken under the same optical configuration and presented with the same maximum/minimum intensity. Red channel, signal from HA antibody; green channel, signal from free GFP. Scale bar, 10 µm. **(F)** Summary data from Panels **(C–E)**. *Y* axis represents membrane intensity in arbitrary fluorescent units. Comparison between treated and untreated cells was caried out using Student’s t-test.

Rescuing the membrane localization and/or function of ion channel mutations, which are degraded by ERAD, with proteasomal degradation inhibitors or by modifying levels of protein ubiquitination have been demonstrated by several independent studies (Rusconi et al., 2007; Arlow et al., 2013; Mohanraj et al., 2019; Kanner et al., 2020). Given that the protein turnover of both S217L and G2029R Piezo1 can be influenced by ALLN treatment, we asked if ALLN also affected the membrane localization of these two mutants. We conducted live cell labeling by overexpressing a Piezo1 construct that contains an extracellular HA tag (Piezo1-897-HA) integrated into a pIRES2-EGFP vector. The HA tag was inserted within an extracellular loop previously shown to be amenable to the insertion of a Myc tag in a similar region in mouse Piezo1 (Coste et al., 2015). Indeed, we tested the function of human Piezo1-897-HA using cell-attached patch clamping and the peak currents elicited in response to negative pressure from Piezo1-897-HA were comparable to those elicited by WT Piezo1 when they were overexpressed in Piezo1^−/−^ HEK293T, indicating the function and localization of Piezo1-897-HA is similar to WT (Figure 4A). We then generated S217L and G2029R mutants based on the same construct by site-directed mutagenesis. Both mutants behaved differently from WT; the membrane labeling intensity was much lower than the wild-type Piezo1. Importantly, upon ALLN treatment, membrane intensity increased by more than 9 folds for G2029R but not for S217L Piezo1 (Figures 4B–F). Our data indicated that membrane localization of G2029R Piezo1 can be partially rescued by the proteasomal inhibitor ALLN.

### Effect of ALLN Treatment on Mechanically-Evoked Currents

**FIGURE 5:**
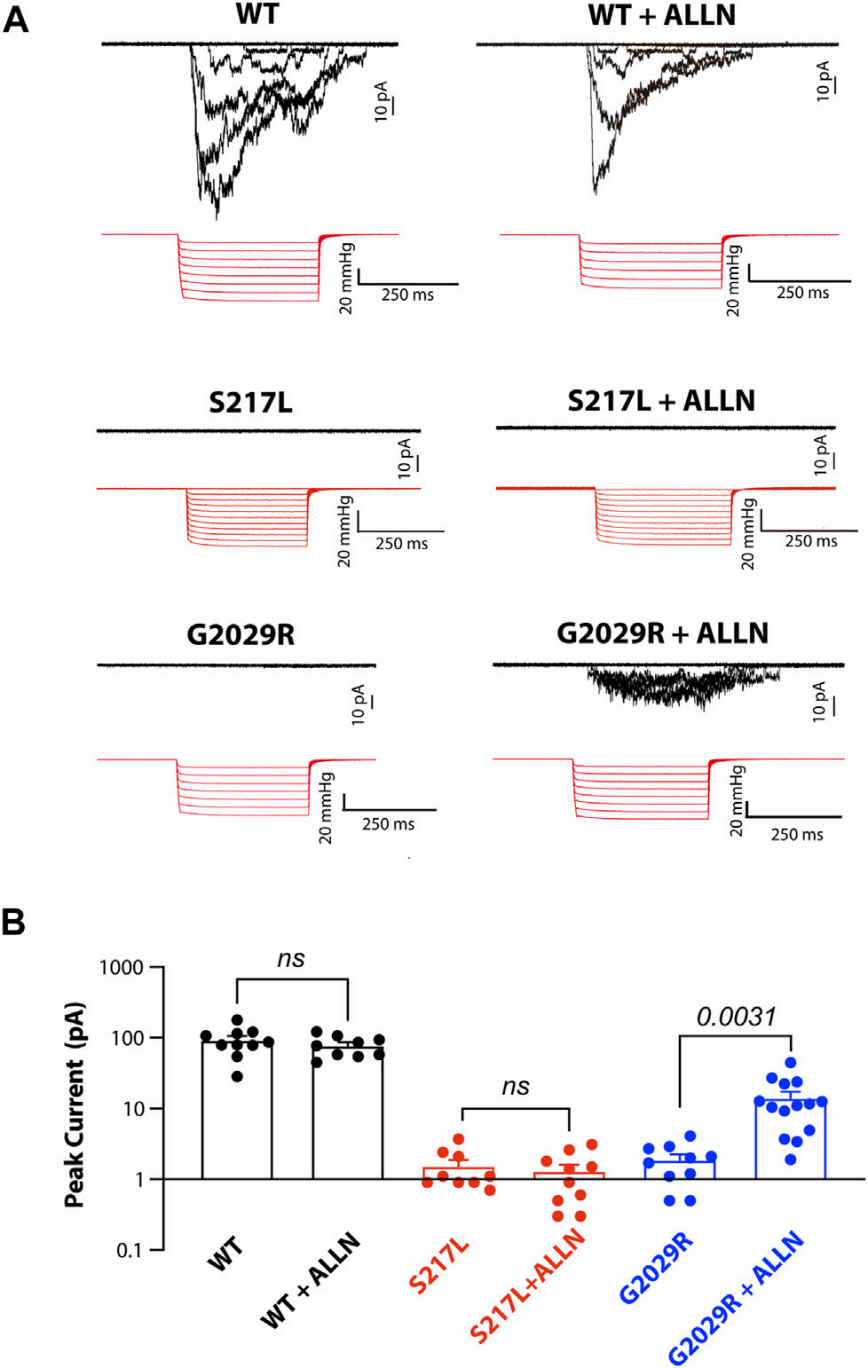
Mechanically-evoked G2029R currents are increased by ALLN treatment. **(A)** Piezo1^−/−^ HEK293T cells overexpressing WT or mutant Piezo1 were treated with or without ALLN for 10–15 h before cell-attached patch clamping. Representative traces are shown. **(B)** Summary data from panel **(A)** illustrating the peak currents elicited from cell-attached patches from Piezo1^−/−^ HEK293T cells overexpressing WT or mutant Piezo1 with or without ALLN treatment. *p* values determined using Student’s t-test to compare treated and untreated cells.

Given that ALLN increased the membrane targeting of G2029R Piezo1 we examined whether the increased membrane localized G2029R Piezo1 correlated with an increase in mechanically evoked currents. We therefore conducted cell-attached patch clamping with Piezo1^−/−^ HEK293T cells overexpressing the WT or mutant Piezo1. We first examined the mechanosensitive currents from WT Piezo1, with and without ALLN treatment. The peak currents displayed only minor differences when comparing between the two groups, suggesting ALLN did not influence WT Piezo1 channel activity (Figures 5A,B). We then studied the electrophysiological behavior of S217L and G2029R Piezo1 with and without ALLN treatment. Consistent with our live labeling data, both ALLN untreated or treated S217L Piezo1 did not respond to mechanical stimuli, while the peak currents of G2029R Piezo1 increased by more than 7.5-fold after ALLN treatment (Figures 5A,B). This data suggested ALLN was able to increase both the membrane localization and mechanically evoked currents from heterologously expressed G2029R Piezo1. Furthermore, this compounds the idea that the mis-localization and increased turnover rate of G2029R Piezo1 serves as a major reason for its loss-of-function.

## Discussion

*PIEZO1* variants have been linked to a number of pathologies (Zarychanski et al., 2012; Albuisson et al., 2013; Bae et al., 2013; Fotiou et al., 2015; Lukacs et al., 2015; Faucherre et al., 2020). The mutations S217L and G2029R are loss-of-function disease-linked *PIEZO1* variants, identified by whole-exome sequencing (Lukacs et al., 2015; Faucherre et al., 2020). Specifically, S217L has been liked to bicuspid aortic valve while G2029R contributes to generalized lymphatic dysplasia. In combination with evidence that loss-of-function *PIEZO1* variants are linked to varicose veins (Faucherre et al., 2020) it seems clear that Piezo1 has an emerging role in valvular formation and function in a number of settings (Nonomura et al., 2018; Faucherre et al., 2020). However, we are only beginning to understand the exact molecular mechanisms underlying the molecular dysfunction of these variants and other disease-linked *PIEZO1* variants.

Loss-of-function variants of ion channels are likely to arise due to two broad mechanisms: 1) aberrant localization or 2) gating defects (potentially increased inactivation or reduced force sensitivity in the case of Piezo1 channels). Previous work points towards defective trafficking of G2029 R (Lukacs et al., 2015) but that S217L had normal membrane localization (Faucherre et al., 2020). We showed that both S217L and G2029R displayed reduced N-glycosylation (Li et al., 2020) [in addition to other trafficking defective mutants generated during structure-function studies (Vero Li et al., 2021)] similar to trafficking defective disease causing variants in ion channels such as K_v11.1_, which causes long QT syndrome type 2 (Vandenberg et al., 2012). Herein we provided further insight into the molecular mechanisms by which these mutations influence Piezo1 function.

First, we observed that both S217L and G2029R had increased protein turnover rates when compared to WT Piezo1 protein. Using a standard cycloheximide treatment strategy to inhibit new protein synthesis it was immediately clear that both mutants were less stable at the protein level. To begin to understand the mechanism of this reduced stability and increased turnover rate, that we had identified previously in mutants with multiple N-linked glycosylation sites mutated (Li et al., 2020), we looked at Piezo1 ubiquitination. We showed that both loss-of-function mutations (S217L and G2029R) underwent increased ubiquitination. This suggested that the degradation of these mutants was likely driven by the proteasome. To test this hypothesis, we treated HEK293T cells expressing Piezo1 with the broad-spectrum proteasome inhibitor ALLN. Consistent with our ubiquitination data ALLN significantly increased the protein levels of both S217L and G2029R. We also showed that the clinically used, and more specific, proteasome inhibitor bortezomib (Chen et al., 2011) at nM concentrations could also reduce the turnover rate of G2029R and boost protein levels. In contrast it seemed the primary route for the degradation of WT Piezo1 in a heterologous expression system involved a lysosomal pathway that was inhibited by NH_4_Cl.

The degradation of proteins through the proteasome is a common route for the disposal of misfolded proteins. Our imaging in HeLa cells showed that both S217L and G2029R exhibited significant retention in the ER consistent with a trafficking defect and our previous data (Li et al., 2020). Although at first glance the level of co-localization with ER tracker in HeLa cells seems not to quantitatively match the differences in current density measured using patch clamping it is important to note that co-localization of ER tracker and WT Piezo1 is expected as the WT protein needs to transit through ER prior to reach its destination at the plasma membrane. When we probed the membrane localized pool of these mutants using membrane biotinylation we did however see some membrane labelling of S217L and G2029R (consistent with the incomplete co-localization of ER tracker and Piezo1 mutants in HeLa cells) but this was significantly reduced compared to WT Piezo1. Some level of membrane labelling is consistent with the immunogold labelling we previously carried out to look at subcellular localization of these mutant proteins (Li et al., 2020). This G2029R data also fits with the small whole-cell indentation currents documented in the first paper implicating G2029R in disease (Lukacs et al., 2015). Altogether these data provide strong support that both S217L and G2029R are largely retained in the ER. The lack of normal trafficking and membrane localization of S217L and G2029R therefore likely provides a significant contribution to the loss-of-function phenotype of these two mutants. However, the residual amount of membrane localised S217L in particular when combined with the complete abolition of stretch-activated currents means we cannot completely rule out an additional gating phenotype in this variant.

Several strategies have been employed to rectify trafficking defects in ion channels that lead to channelopathies as a potential route to therapies. This includes a recent report that the reversal of ubiquitination may act as a potential broad-spectrum strategy to rectify channel trafficking defects (Kanner et al., 2020). We previously used N-linked glycosylation as a surrogate of membrane trafficking and tried to use two widely used strategies, low temperature and a chaperone (Anderson et al., 2006; Robertson and January 2006; Anderson et al., 2014), to improve the trafficking of G2029R. Neither strategy was successful (Li et al., 2020). In the current study given the robust effects of ALLN on mutant Piezo1 protein levels we next asked the question of whether proteasome inhibition could rescue trafficking of Piezo1 mutants as shown for other ion channels (Wang et al., 2018).

**FIGURE 6:**
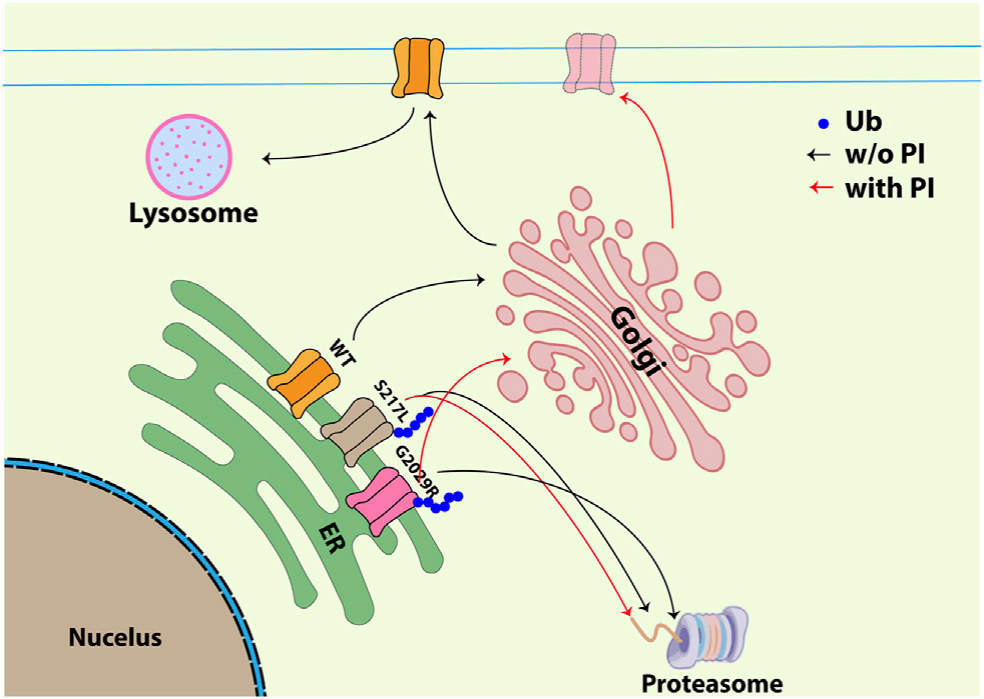
Schematic figure illustrating the trafficking pathway for WT, S217L and G2029R with or without ALLN. WT Piezo1 transits from ER to plasma membrane and is finally degraded through membrane-protein internalization and lysosome dependent degradation. Unlike the WT Piezo1, both S217L and G2029R Piezo1 go through ubiquitination and proteasome dependent degradation. Treatment with the proteasomal inhibitor ALLN did not influence the membrane localization of S217L, while G2029R was partially rescued exhibiting increased membrane labelling and increased amplitudes of mechanically-evoked currents. Black arrow indicates the trafficking pathway for all Piezo1 proteins without ALLN treatment; red arrow indicates the trafficking pathway for S217L and G2029R Piezo1 after ALLN treatment (Ub-ubiquitin, P/I-proteasomal inhibition).

We showed using both a live labelling approach, similar to previous studies (Lukacs et al., 2015; Saotome et al., 2018), and patch clamp electrophysiology that ALLN not only could increase the membrane labelling of G2029R but could also cause a modest increase in the cell-attached mechanically-evoked currents (Figure 6). The S217L live cell labelling and stretch-activated currents were not affected by ALLN even though it did slow the rate of protein turnover. This is not surprising given that for K_v11.1_ there is a continuum where some channel mutations can be rescued by low temperature, some by molecular chaperones, some by both approaches and some are resistant to these strategies (Anderson et al., 2006; Smith et al., 2013; Anderson et al., 2014). It is therefore highly likely that Piezo1 mutants may also display this behaviour.

In summary, here we showed that two loss-of-function *PIEZO1* variants linked to different diseases show reduced stability at the protein level which correlates with increased ubiquitination. For both mutants, proteasome inhibition increased the protein amount implicating proteasomal degradation in their turnover. For one of these mutants (G2029R) we could boost the membrane targeting and stretch-activated currents via proteasomal inhibition. This suggests that in the future utilizing proteasomal inhibitors in use in the clinic in combination with a precision medicine-based approach we may be able to rectify the mis-localization and reduced function of Piezo1 channels associated with a subset of Piezo1-channel mediated channelopathies.

## Data Availability

The raw data supporting the conclusion of this article will be made available by the authors, without undue reservation.
